# Muscle Dysmorphic Disorder Inventory (MDDI): Validation of a German version with a focus on gender

**DOI:** 10.1371/journal.pone.0207535

**Published:** 2018-11-16

**Authors:** Almut Zeeck, Viola Welter, Hasan Alatas, Tom Hildebrandt, Claas Lahmann, Armin Hartmann

**Affiliations:** 1 Department of Psychosomatic Medicine and Psychotherapy, Medical Center–University of Freiburg, Faculty of Medicine, University of Freiburg, Freiburg, Germany; 2 Eating and Weight Disorders Program Icahn School of Medicine at Mount Sinai, New York, New York, United States of America; University of Lleida, SPAIN

## Abstract

Muscle dysmorphia (MD) is a condition that is characterized by body image disturbance, a drive for muscularity and excessive exercising. It leads to considerable functional impairment. Most previous studies focused on male samples. The study aimed to validate a German version of the Muscle Dysmorphic Disorder Inventory (MDDI) in order to make the instrument available in German speaking countries. We further aimed to explore for gender differences in the MDDI factors (measurement invariance) and to assess the relationship between MD and positive dimensions of body experience as well as exercise dependence. 394 participants (53% females, mean age 24.3 years) took part in an internet-based survey. The three-factor structure of the English version of the MDDI was replicated, independent of gender (multi group CFA; Base model *TLI* = .961; *CFI* = .970). Cronbach´s alpha was .81-.84 for the subscales and .75 for the MDDI total score. MD was associated with exercise dependence and negatively correlated with dimensions of positive body experience, which can be considered relevant for satisfying relationships and a positive sense of self: e.g. body contact and sexual fulfillment. Men and women showed differences in two subscales of the MDDI (appearance intolerance, drive for size). Testing for measurement invariance resulted in weak invariance: Equivalent factor structure for men and women, but significantly different loadings and coefficients. No statistically significant difference in the MDDI total score was found. The findings suggest good psychometric properties of the German version of the MDDI. Future studies should address the question of cut-off scores and norms for different samples as well as a possible overlap between MD and eating disorder psychopathology in women.

## Introduction

Muscle dysmorphia (MD) is a condition that “is characterized by a fear of being too small, and by perceiving oneself as small and weak, even when one is actually large and muscular.” [1]. It is reported to be most prevalent in weightlifters and bodybuilders [2]. MD encompasses a psychological, behavioral and social dimension. On the psychological level, individuals with MD show a pathological pursuit of muscularity, including a preoccupation with their appearance and body image disturbance: Although they are usually leaner and more muscular than others, they feel small and try to hide their body [3]. Showing their body leads to intense shame and embarrassment [4]. On a behavioral level, MD is characterized by excessive hours of exercising, disordered eating practices and often steroid use [5,6]. Life time prevalence rates of mood disorders, anxiety disorders and eating disorders in individuals with MD are significantly higher compared to controls [2]. Overall, MD is a disabling condition that—on a social level—leads to considerable impairment in work functioning and a withdrawal from social contacts [7]. Nevertheless, MD often remains undetected [2].

Pope et al. [3] proposed diagnostic criteria for MD, which are still valid today. However, although MD was included in the DSM-5 as a subtype of body dysmorphic disorder [8], there is still no consensus on this decision. It remains a topic of debate, if MD should be categorized as a body dysmorphic disorder, an eating disorder, a behavioral addiction or an obsessive-compulsive disorder [6,9,10].

It is uncontroversial that exercise psychopathology is a key symptom in MD. Therefore, one could expect a close relationship between MD and exercise dependence, as excessive hours of exercising, an increase in negative affect when missing exercise sessions and an impact on social functioning will be overlapping features [11]. Most definitions of exercise dependence are oriented on criteria of addiction and include the following symptoms: Tolerance, withdrawal symptoms when stopping to exercise, continuance despite negative consequences, time (spending large amounts of time exercising), reduction in other activities, lack of control and intention effects (exercising longer than expected) [12]. Exercise dependence is not characterized by body image disturbance or appearance intolerance, which are key symptoms in MD.

Authors criticize the underrepresentation of males in studies on body image disturbance and disordered eating [6,13], because an over-evaluation of shape and weight is not limited to females and increasingly present in men [13,14]. The internalization of an ideal body figure as well as social body comparison were found to be associated with a drive for muscularity in men and may increase overall body dissatisfaction [15,16]. In contrast to women, men seem to be preoccupied with a drive for muscularity and body composition (leanness) rather than thinness [17].

Dos Santos Filho et al. [9] conducted a systematic review on studies in MD and identified 34 articles. The samples mainly consisted of male bodybuilders, weightlifters or college students. Prevalence rates of MD ranged from over 50% in studies assessing highly selective groups like competitive bodybuilders to about 6–7% in primarily male college students [18–20]. However, it is important to note that most studies used screening instruments (self-report) and not a clinical interview. Therefore, it can be assumed that clinically relevant MD is less common and probably a rare condition in the general population. Only six articles in this review reported on samples including females [9]. Furthermore, more than half of the studies were conducted in the U.S., and most of them included sample sizes of less than 100 participants.

A prerequisite for research in the field are validated instruments which are translated in several languages. Validated instruments are also useful for screening purposes in clinical practice. There are instruments for the measurement of a drive for muscularity like the DMS (Drive for Muscularity Scale; [17,21]) and instruments that aim to assess features of MD more specifically, based on Pope et al.´s criteria [3]. Instruments for the assessment of MD comprise the MDSQ (Muscle Dysmorphia Symptom Questionnaire), the MASS (Muscle Appearance Satisfaction Scale), the MDQ (Muscle Dysmorphia Questionnaire), the MDI (Muscle Dysmorphic Inventory) and the MDDI (Muscle Dysmorphic Disorder Inventory; [22]). The MDI and the MASS were found to be the most used instruments in a recent systematic review and meta-analysis, followed by the MDDI [23]. However, MASS and MDI do not address the functional impairment aspect, which is important to evaluate the pathological dimension of MD and one diagnostic criterion. Therefore, we chose the MDDI for translation, which improved the MDI by adding a scale that measures functional impairment. The MDDI was already validated in Italian language [24,25]. The Italian version revealed the same factor structure as the original version and showed good convergent and divergent validity. Cronbach´s alpha was between *α* = .80 and *α* = .85 for the total score and two subscales. However, one subscale (appearance intolerance) showed a low level of internal consistency (*α* = .45) [25].

## Aims of the study

We aimed to validate the German version of the MDDI in order to make the instrument available for the assessment of muscular dysmorphia (MD) in German-speaking countries. Secondly, we aimed to evaluate the influence of gender (factor structure including measurement invariance, psychopathological features of MD in men and women). Finally, and as part of the validation process we intended to analyze the relationship between psychopathological features of MD and different dimensions of positive body experience (e.g. body acceptance, body contact, vitality, sexual fulfillment) as well as exercise dependence.

We expected features of MD and especially a drive for muscularity to be significantly more prevalent in males compared to females, although we assumed MD to be also present in women. In terms of convergent validity, we hypothesized that there will be a) a positive correlation between the MDDI total score and exercise dependence in both, men and women and b) a negative correlation between appearance intolerance and the acceptance of one´s own body. Finally, we assumed a negative correlation between MD (MDDI total score) and further dimensions of body experiences, which can be assumed to contribute to quality of life and satisfying relationships (body contact, sexual fulfillment, vitality), independent of gender.

## Materials and methods

### Translation of the MDDI

The translation was conducted according to the standards for the translation of instruments [26,27]. The items of the MDDI were translated into German by the first and third author (AZ, HA). Two words were difficult to translate in such a way that they capture the exact meaning in the context of body experience in German: “big” and “small”. Their translation was discussed in detail within the research group. In a next step, items were back-translated by a native speaker. In case of a deviance, the German translation was discussed and adapted. In a final step, the back-translation was presented to the developer of the MDDI (TH), who approved the final version (for items see Table 1).

**Table 1:** Items of the MDDI: English and German version.

English (original version): Item	German: Item	Subscale
I01. I think my body is too skinny/slender.	1. Ich finde meinen Körper zu schmächtig	DS
I02. I wear loose clothing so that people can't see my body.	2. Ich trage weite Kleidung, sodass Menschen meinen Körper nicht sehen können	AI
I03. I hate my body.	3. Ich hasse meinen Körper	AI
I04. I wish I could be heavier.	4. Ich wünschte mir, ich könnte kräftiger werden	DS
I05. I find my chest to be too small.	5. Ich finde meinen Oberkörper zu schmächtig	DS
I06. I think my legs are too thin.	6. Ich finde meine Beine zu dünn	DS
I07. I feel like I have too much body fat.	7. Ich fühle mich, als wenn ich zu viel Körperfett habe	AI
I08. I wish my arms were stronger.	8. Ich wünschte mir, meine Arme wären kräftiger	DS
I09. I am embarrassed to let people see me without a shirt or t-shirt.	9. Ich schäme mich, mich Menschen ohne Hemd / T-Shirt zu zeigen	AI
I10. I feel anxious when I miss one or more days of exercise.	10. Ich fühle mich unruhig / ängstlich, wenn ich einen oder mehrere Trainingstage verpasse	FI
I11. I cancel social activities with friends (e.g. watching football, invitations to dinner, going to the movie theater, etc.) because of my workout/exercise schedule.	11. Ich schlage soziale Aktivitäten (z.B. Fußballspiele schauen, Essenseinladungen, ins Kino gehen, etc.) mit Freunden aufgrund meines Trainingsplans aus	FI
I12. I feel depressed when I miss one or more days of exercise.	12. Ich fühle mich niedergeschlagen, wenn ich einen oder mehrere Trainingstage verpasse	FI
I13. I miss opportunities to meet new people because of my workout schedule.	13. Ich lasse mir aufgrund meines Trainingsplans Chancen entgehen, neue Menschen kennenzulernen	FI

Note: DS = drive for size, AI = appearance intolerance, FI = functional impairment

### Sample

Participants were recruited through interest groups (fitness, bodybuilding) in social media using Lime Survey (www.limesurvey.org). The survey could be processed on an online platform without entering personal data (anonymously). First, an information page on the aims of the study was presented, followed by questions on socio-demographic data and self-report questionnaires. Inclusion criteria were an age between 18 and 45 years and being physically active: Participants should train at least three times a week in a fitness gym. The study was approved by the local ethics committee (University of Freiburg; No 17/14).

Overall, 394 individuals took part in the survey (for sample description see Table 2).

**Table 2:** Sample description.

**Variable**	**Values / metric**	***M (SD) / % (N)***
**Age**	years	24.3 (5.2)
**Gender**	female	53% (209)
	male	47% (184)
	unknown	<1% (1)
**Nationality**	German	88.8% (350)
	Other	11.2% (44)
**Partnership**	Yes	45.5% (180)
**Education**	University	35.4% (140)
	Student, in education	27.8% (110)
	Other	19.7% (78)
	Unknown	17.1% (66)
**Occupation**	Full time	31.2% (123)
	In education	28.7% (113)
	Working part time or occasionally	19.0% (75)
	Unemployed	1.0% (4)
	Other (housewife, retired, disabled, etc.)	20.1% (79)
	

### Instruments

#### MDDI

The Muscle Dysmorphic Disorder Inventory [22] is a 13 item questionnaire, which showed good internal consistency (total score: *α* = .81) and good to excellent retest-reliability (*r* = .87). It was based on the muscle dysmorphia inventory MDI [28], improving this instrument by adding an additional dimension (functional impairment). The items form three subscales (drive for size (DS), appearance intolerance (AI), functional impairment (FI)). Cronbach´s alpha for the subscales was *α* = .85 for DS, it was *α* = .77 for AI and *α* = .80 for FI. Items (see Table 1) can be rated on a five-point Likert scale with responses ranging from 1 (never) to 5 (always). A total score can be derived by the sum of the subscales. A threshold value of > 39 points was proposed [29] and used in a previous study (e.g. [24]).

#### DKB-35

The Dresden Body Image Inventory [30] is a German questionnaire (self-rating) for the assessment of cognitive as well as affective components of body image. It comprises 35 items and five subscales: Vitality, body- acceptance, self-enhancement, body contact and sexual fulfillment. Construct validity and differential validity were demonstrated as well as retest reliability [31]. Internal consistency varied between Cronbach´s *α* = .76 and *α* = .91.

#### EDS-D

The Exercise Dependence Scale ([12]; German version: [32]) is a self-report instrument with 21 items, which was developed to measure exercise dependence. There are seven subscales, oriented on criteria for addiction (tolerance, withdrawal effects, continuance, lack of control, reduction in other activities, time, intention). The items are rated on a 6-point Likert scale. The EDS showed good validity and internal as well as test-retest reliability [33]. Criteria were defined to distinguish three groups: Individuals “at risk for exercise dependence”, which have a score of 15 or more on at least three subscales, individuals with some symptoms (“nondependent-symptomatic”: showing a score of 7 or more on at least three subscales and not being classified as “at risk”) and a “non-dependent-asymptomatic” group.

#### Questionnaire on sports behavior [34]

The quantity of exercise was assessed with the German ‘‘Fragebogen zum Sportverhalten” by asking for type of sports activity, frequency (per month) and duration (per episode). For analysis, the minutes of sports activity per week were calculated (physical exercise index).

### Data analysis

Confirmatory factor analyses (CFAs, ML estimator) were computed with R (V3.1.4), the lavaan library (V0.5–23.1097), and its embedded procedures (esp. cfa, measurementInvariance). The fit of the factor structure was determined by the criteria of Hu and Bentler [35], where adequate fit is indicated by *CFI* and *TLI* > .95, *RMSEA* < .06 and *SRMR* < .08. To examine measurement invariance between men and women a multi group CFA was conducted. Measurement invariance between male and female samples was examined by *Chi*^*2*^ and *CFI* difference statistics. In order to estimate gender specific models, two separate CFAs were computed. The online survey required listwise complete data for all MDDI-Items. One subject did not indicate his/her gender, thus reducing the available *N* for subgroup analyses by one. Descriptive statistics and difference tests of subsamples were computed with SAS-JMP (V.10).

## Results

### MDDI scores: Descriptive statistics

There was no significant difference in the MDDI total score when comparing men and women. However, men had significantly higher scores on the scale “drive for size” (DS) compared to women, while women showed significantly higher scores for “appearance intolerance” (AI). See Table 3 for item and factor scores as well as comparisons.

**Table 3:** Descriptive statistics of items and scores.

Item/Score	Factor		*α* **	All; *N* = 394					Male; *N* = 184				Female; *N* = 209				Wilcox*
		Corrected item total correlation		*M*	*SD*	*Min*		*Max*	*M*	*SD*	*Min*	*Max*	*M*	*SD*	*Min*	*Max*	*P<*
I01 Body too skinny	DS	.80	.79	2.14	1.07	1		5	2.56	1.02	1	5	1.78	0.98	1	5	.001
I02 Wear losse clothing	AI	.81	.79	2.11	1.06	1		5	1.75	0.92	1	5	2.42	1.08	1	5	.001
I03 Hate my body	AI	.83	.78	2.07	1.05	1		5	1.75	0.96	1	5	2.35	1.04	1	5	.001
I04 Wish to be havier	DS	.81	.79	2.82	1.22	1		5	3.37	1.10	1	5	2.33	1.12	1	5	.001
I05 Chest too small	DS	.82	.78	2.12	1.14	1		5	2.53	1.09	1	5	1.76	1.05	1	5	.001
I06 Legs too thin	DS	.70	.83	1.78	1.06	1		5	2.31	1.15	1	5	1.31	0.72	1	5	.001
I07 Too much body fat	AI	.78	.81	3.45	1.18	1		5	3.18	1.21	1	5	3.68	1.11	1	5	.001
I08 Arms should be stronger	DS	.75	.81	2.73	1.20	1		5	3.18	1.14	1	5	2.34	1.11	1	5	.001
I09 Embarrassed letting people see me	AI	.84	.76	2.23	1.24	1		5	1.83	1.11	1	5	2.59	1.24	1	5	.001
I10 Anxious if not exercising	FI	.77	.79	3.23	1.18	1		5	3.14	1.22	1	5	3.30	1.14	1	5	.185
I11 Cancel social activities	FI	.83	.77	2.56	1.05	1		5	2.48	1.07	1	5	2.62	1.03	1	5	.150
I12 Depressed if not exercising	FI	.80	.75	3.31	1.11	1		5	3.22	1.16	1	5	3.39	1.06	1	5	.137
I13 Not meeting new people	FI	.81	.76	2.33	1.09	1		5	2.26	1.06	1	5	2.39	1.10	1	5	.238
DS Drive for Size			.84	11.58	4.43	5		25	13.95	4.11	5	25	9.53	3.58	5	23	.001
AI Appearance Intolerance			.83	9.86	3.69	4		20	8.50	3.31	4	20	11.03	3.61	4	20	.001
FI Functional Impairment			.81	11.39	3.54	4		20	11.08	3.61	4	20	11.68	3.46	4	20	.068
MDDI TOTAL Score				32.83	7.37	14		54	33.53	7.71	14	54	32.25	7.01	17	54	.062

Note: Labels of items are abbreviations; gender: missing data for one case

* Wilcoxon Rank Sum Test for significant difference between genders, all distributions slightly but significantly different from normal distribution

** standardized alphas for items “if item removed”

Scoring keys: *DS* = *I*1+*I*4+*I*5+*I*6+*I*8;*AI* = *I*2+*I*3+*I*7+*I*9;*FI* = *I*10+*I*11+*I*12+*I*13;*TOTAL* = *DS*+*AI*+*FI*

### Confirmatory factor analysis

**Fig 1:**
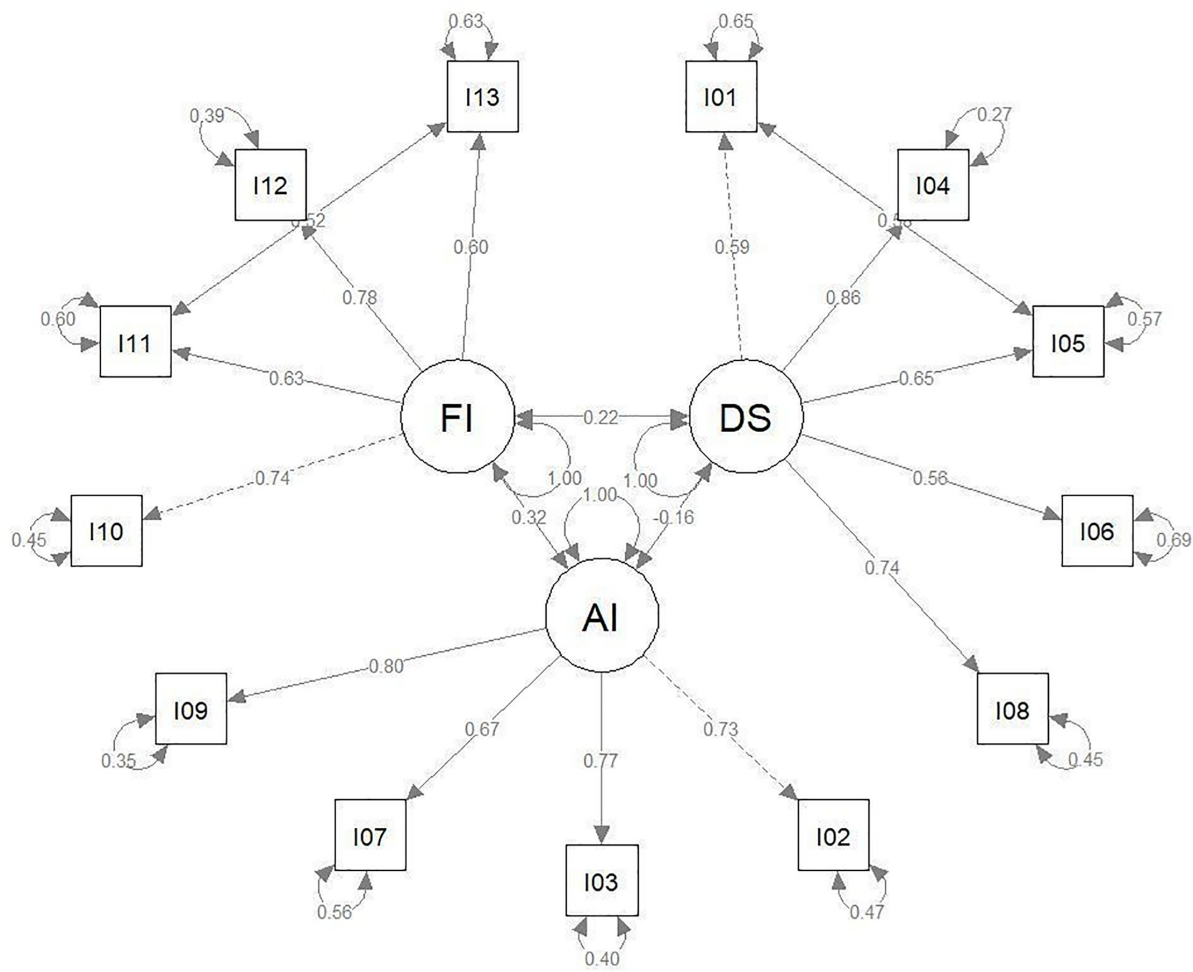
R-Code of MDDI factor model. model.MDDI <—'.+ DS = ~ I01 + I04 + I05 + I06 + I08.+ AI = ~ I02 + I03 + I07 + I09.+ FI = ~ I10 + I11 + I12 + I13.+ DS ~~ AI.+ DS ~~ FI.+ AI ~~ FI.+ I01~~I05.+ I11 ~~ I13'.

The factorial model for the CFA (ML estimator) relied on the factorization of Hildebrandt et al. [22], which suggests three independent factors (DS, AI, FI). This model yielded insufficient fit indices, where modification indices indicated correlated errors of the Item pairs (I01 = “I think my body is too skinny / slender, I05 = “I find my chest to be too small”; I11 = “I cancel social activities with friends because of my exercise schedule, I13 = “I miss opportunities to meet new people because of my workout schedule”). The final model took care of the error covariance. In both cases the correlated errors occur within one factor. There are no multiple factor loadings or correlated errors between factors. Therefore we evaluated the correlated errors as a minor disturbance of the theoretical model fit (see Fig 1).

The model fitted the data sufficiently well (estimator = *ML*). The same model was applied to the subsamples of male and female subjects. The model was very similar in both subsamples and the total sample (see Table 3). The Chi^2^-test was always significant, while *CFI*, *TLI*, *RMSEA* and *SRMR* indicated good or sufficient fit (*CFI*, *TLI* > .95; *RMSEA* < .06; *SRMR* < .08). The fit indices of all three models are listed in Table 4. The parameters of the base model including all available cases are shown in Fig 1 and Table 5.

**Table 4:** Fit indices of the CFA models.

Sample	*Chi^2^; df; p<*	*TLI*	*CFI*	*RMSEA (CI90) p<*	*SRMR*
**Total sample**	121.8; 60; .001	.961	.970	.051 (.038 | .064) .428	.048
**Male sample**	87.7; 60; .011	.957	.967	.072 (.025 | .072) .478	.065
**Female sample**	89.6; 60; .008	.960	.969	.048 (.25 | .068); .528	.055

Note: criteria of good fit = *TLI*, *CFI* > 0.95; *RMSEA* < 0.06; *SRMR* < 0.08, *Chi*^*2*^ = n.s.

**Table 5:** Parameters of the fitted model (whole sample).

Relation			*Est*	*SE*	*CI*.*lower*	*CI*.*upper*
**DS**	**= ~**	**I01**	1.000	0.000	1.000	1.000
**DS**	**= ~**	**I04**	1.657	0.150	1.364	1.951
**DS**	**= ~**	**I05**	1.179	0.077	1.028	1.329
**DS**	**= ~**	**I06**	0.939	0.107	0.729	1.148
**DS**	**= ~**	**I08**	1.408	0.132	1.149	1.667
**AI**	**= ~**	**I02**	1.000	0.000	1.000	1.000
**AI**	**= ~**	**I03**	1.049	0.077	0.898	1.200
**AI**	**= ~**	**I07**	1.020	0.085	0.853	1.187
**AI**	**= ~**	**I09**	1.289	0.092	1.108	1.469
**FI**	**= ~**	**I10**	1.000	0.000	1.000	1.000
**FI**	**= ~**	**I11**	0.764	0.073	0.621	0.907
**FI**	**= ~**	**I12**	0.999	0.086	0.830	1.168
**FI**	**= ~**	**I13**	0.755	0.075	0.607	0.902
**DS**	**~~**	**AI**	-0.079	0.030	-0.138	-0.020
**DS**	**~~**	**FI**	0.120	0.036	0.049	0.191
**AI**	**~~**	**FI**	0.213	0.046	0.124	0.302
**I01**	**~~**	**I05**	0.432	0.051	0.332	0.533
**I11**	**~~**	**I13**	0.363	0.051	0.263	0.463
**I01**	**~~**	**I01**	0.748	0.060	0.630	0.866
**I04**	**~~**	**I04**	0.400	0.063	0.277	0.524
**I05**	**~~**	**I05**	0.734	0.062	0.613	0.856
**I06**	**~~**	**I06**	0.780	0.061	0.660	0.899
**I08**	**~~**	**I08**	0.645	0.063	0.522	0.768
**I02**	**~~**	**I02**	0.531	0.048	0.436	0.625

Note: DS = drive for size, AI = appearance intolerance, FI = functional impairment; NA = not available; *CI* = confidence interval; *p* = .95 for *CI*.*lower* and *CI*.*upper p* = .95; I01 –I13 = item numbers of the MDDI; *Est* = estimate, *SE* = standard error

### Measurement invariance between male and female samples

Testing the measurement invariance of the three models in a multi group CFA showed a weak invariance, as the loadings were not significantly different (see Table 6, tests of ΔChi^2^) between the subsamples of male and female subjects. Significant differences were found for intercepts and means.

**Table 6:** Measurement invariance.

Fit	*Df*	*AIC*	*Chi^2^*	*ΔChi^2^*	*Df diff*	*P<*	*CFI*	*ΔCFI*	*RMSEA*	*ΔRMSEA*
**configural**	122	13677	235.5	-	-	-	.937	-	.069	-
**loadings**	132	13671	249.6	14.0	10	.172	.935	.002	.067	.001
**intercepts**	142	13683	281.7	32.1	10	.001	.923	.012	.071	.003
**means**	145	13837	441.6	159.9	3	.001	.836	.087	.102	.031

Note: fit.configural = base model; fit.loadings = testing for differences in factor structure (n.s.); fit.intercepts = testing for differences in item means (sign. gender differences); fit.means = testing for differences in latent variable means.

The incremental change of Chi-Square values and its significance test (see Table 4) show that the loadings are not significantly different for men and women. In other words, the factor structure is invariant. However, the intercepts and means differ significantly. This corresponds to the significant difference found in the factor scores. As already shown above with the item statistics (Table 3), the main differences between male and female subjects were found in the factors appearance intolerance (AI; females > males) and drive for size (DS; males > females).

### Reliability

Cronbach´s alpha for the DS subscale was *α* = .84, Cronbach´s alpha for the AI subscale was *α* = .83, for the FI subscale it was *α* = .81 and for the total score Cronbach´s alpha was *α* = .75.

### Individuals “at risk” for muscle dysmorphia

Using a cut-off of > 39, 20.3% of individuals (80/394) were classified as “at risk” for muscle dysmorphia. In the “at risk” group were more men than women (46 vs. 34; *Chi^2^* = 4.602; *df* = 2; *p* < .032).

### Correlations with body experience (DKB-35) and exercise pathology (EDS)

Results for correlations with the EDS-D total score are shown in Table 7, correlations with DKB-35 scales in Table 8. The EDS-D total score showed significant correlations with all MDDI scales (small correlations according to Cohen *r* < .3 for associations with drive for size (DS) and appearance intolerance (AI), large correlations (*r* > .5) with functional impairment (FI) and the total score).

**Table 7:** Correlations of MDDI factors with the EDS-D total score.

ID	Variable	*N*	*M*	*SD*	[1]	[2]	[3]	[4]	[5]
**[1]**	**MDDI_DriveForSize**	394	11.58	4.43	1	- .14	.20	.63	.13
**[2]**	**MDDI_AppearanceIntolerance**	394	9.86	3.69		1	.26	.54	.27
**[3]**	**MDDI_FunctonalImpairment**	394	11.39	3.54			1	.73	.63
**[4]**	**MDDI_Total**	394	32.83	7.37				1	.52
**[5]**	**EDS_Total**	352	3.08	0.80					1

Note: All correlations sign., *p* < .05; Pearson Corr. Coefficients.

**Table 8:** Correlations of MDDI factors with the DKB-35 scales.

Variable	*M*	*SD*	MDDI DS	MDDI AI	MDDI FI	MDDI Total
**DKB35_1Vitality**	3.88	0.53	-.05	**-.48**	-.02	**-.28**
**DKB35_2BodyAcceptance**	2.23	0.59	.08	**-.83**	**-.24**	**-.49**
**DKB35_3SexualFulfillment**	2.70	0.86	-.02	**-.36**	**-.21**	**-.29**
**DKB35_4SelfEnhancement**	2.24	0.58	.05	**-.34**	.03	-.13
**DKB35_5BodyContact**	3.78	0.72	-.03	**-.35**	**-.21**	**-.29**

Note: All correlations with *p* < .05 in bold letters; *N* = 370

The appearance intolerance (AI) subscale and all scales of the DKB-35 correlated negatively and significantly (medium to large *r*). Functional impairment (FI) correlated negatively with body acceptance, body contact and sexual fulfillment (small *r*). There was no association between the drive for size (DS) subscale and the DKB-35 scales.

### MDDI scores and risk for exercise dependence

32 individuals were classified “at risk for exercise dependence”, 189 as “non-dependent-asymptomatic” and 131 as “non-dependent symptomatic”. The “at risk for exercise dependence” group showed significantly higher values for appearance intolerance (AI) (*F* = 6.387; *df* = 2; *p* < .002), functional impairment (FI) (*F* = 44.727; *df* = 2; *p* < .001) as well as the MDDI total score (*F* = 24.565; *df* = 2; *p* < .001). However, there was no significant difference in drive for size (DS) scores between groups.

## Discussion

We were able to replicate the factor structure of the MDDI in the German version, independent of gender. The model fit is slightly impaired by unexplained correlations of the errors of two pairs of items and a significant Chi^2^-Test, but overall, the limitations are minor and acceptable for a translation and application of the test in another country. However, the invariance of the MDDI factors between genders is only given for the configuration and loadings of the items, not for item means and factor means. This is consistent with the expectation to find different patterns of symptomatology between men and women: According to our hypothesis, men showed a stronger wish for muscularity, while women had more difficulties with tolerating their appearance (hating their body, feeling to have too much body fat and wanting to hide their body). Both aspects were also differentially associated with functional impairment: Appearance intolerance was more relevant for functional impairment in women, while a drive for muscularity was in men. The result is in line with previous findings that men show a stronger drive for muscularity, while women are more preoccupied with thinness [17,36].

Internal consistency was comparable to the original version (Cronbach´s alpha between *α* = .80 and *α* = .85), with only a slightly lower level for the appearance intolerance subscale (AI; *α* = .77). A lower level of internal consistency for the AI subscale was also found in the Italian version.

In our study women and men did not differ in functional impairment and showed a similar total score of the MDDI. However, when using the cut-off of > 39 points, more men (25% vs. 16%) were classified “at risk” than females. Longobardi et al. [24] found a comparable percentage of 25% to be “at risk” for MD in an online survey for validating the MDDI in Italy (male sample). Although the higher prevalence of MD in males is in line with previous research, it seems more important to mention the relatively high percentage of women that were classified “at risk” for MD in our study, although we cannot rule out that this finding is due to an overlap with features of an eating disorder and general body dissatisfaction.

Overall, we think there is the need for further studies on prevalence rates of MD in different samples (athletes, females, community samples), combining screening instruments and clinical interviews. Furthermore, more research is necessary to clarify the relevance of this disturbance for women. This is also important before the background of a change in body ideals: A more athletic and muscular ideal seem to play an increasing role in media-transported ideals for women [37].

We found that the MDDI total score was significantly correlated with the total score of the EDS (exercise dependence). Additionally, the subgroup that was classified “at risk for exercise dependence” showed the highest MDDI total scores, which can be considered an indicator for convergent validity. The correlation between the MDDI and the EDS total score was mainly due to high correlations with the subscale functional impairment. The items of the functional impairment subscale address aspects that coincide with central criteria for exercise dependence: Feeling depressed or anxious when missing exercise sessions, canceling social activities or missing opportunities to meet new people because of exercising. This replicates previous findings that exercise pathology is a key symptom of MD. However, small (although significant) correlations with the two other MDDI-Scores (appearance intolerance, drive for size) also point to differences between both conditions.

In terms of the relationship with dimensions of body experience, appearance intolerance showed a large correlation with low body acceptance. Furthermore, appearance intolerance was associated with difficulties in body contact, less sexual fulfillment and less vitality. These dimensions were also associated with functional impairment. Overall, the findings show that MD is associated with impairments in body experience that can be considered highly relevant for satisfying relationships and a positive sense of self, replicating a previous finding [7]. However, it is unclear if MD leads to difficulties with body contact and sexuality, or if the attempt to attain a powerful body is an attempt to compensate for feelings of inadequacy, vulnerability and a negative self-concept. The latter would be supported by a study of Fabris et al. [38], who found an association between a risk for MD and more insecure (especially avoidant) attachment styles. Insecure attachment styles are characterized by more negative models of self and others, caused by early developmental experiences [39].

One result did not correspond to our assumptions. For the drive for size scale (DS) we found only a small correlation with exercise dependence (EDS total score) and no significant correlations with different dimensions of body experience (DKB-35 scales: vitality, body acceptance, sexual fulfillment, self-enhancement and body contact). This finding is difficult to explain and seems not due to overall low scores on this scale (in our study it was *M* (mean) = 14.0 for men; in a study including male competing body-builders it was *M* = 15.4, while the score was *M* = 10.0 for not competing males [25]; in another study the score was *M* = 20.6 in males with MD and 10.6 in males with anorexia nervosa [40]). One possible explanation could be that an urge for muscularity alone is not necessarily a sign of psychopathology. This might only be the case if it is accompanied by a body image disturbance and excessive training.

In summary, the strength of this study on MD is to include both genders and a larger sample. A further strength is to address the relationship between MD symptoms and positive dimensions of body experience as well as the relationship to exercise dependence. A main limitation is that we did not include a measure of eating pathology (however, instruments had to be restricted to assure compliance with an online-survey). Since it was a self-report online-survey, we cannot reliably estimate the percentage of individuals with a clinically relevant MD (structured interviews would have been necessary to address this question). Furthermore, there the risk for a selection bias in a self-selected internet survey.

Overall, the German version of the MDDI can be considered an instrument that can be used in male as well as female samples to assess symptoms of muscle dysmorphia in German speaking countries. However, as the MDDI is a self-report tool, no diagnosis can be derived. For such a purpose, it has to be combined with a clinical interview.

Future studies should address test-retest-reliability to analyze temporal stability of the tool as well as the question of cut-off scores and norms for different samples (males, females, community samples and risk groups), which are relevant to identify individuals in need for further clinical assessment and treatment.
